# Clopidogrel protects against gentamicin-induced nephrotoxicity through targeting oxidative stress, apoptosis, and coagulation pathways

**DOI:** 10.1007/s00210-024-03380-5

**Published:** 2024-09-05

**Authors:** Asmaa A. Akila, Rania A. Gad, Mohamed Gamal El-Din Ewees, Manal Abdul-Hamid, Eman S. Abdel-Reheim

**Affiliations:** 1 Molecular Physiology Division, Department of Zoology, Faculty of Science, Beni-Suef University, Beni-Suef, 62511 Egypt; 2 Department of Pharmacology and Toxicology, Faculty of Pharmacy, Nahda University, Beni-Suef, 62511 Egypt; 3 Cell Biology and Histology Division, Department of Zoology, Faculty of Science, Beni-Suef University, Beni-Suef, 62511 Egypt

**Keywords:** Gentamicin, Clop, Fibrin, Apoptosis, Oxidative stress

## Abstract

Gentamicin (Genta)-induced nephrotoxicity poses a significant clinical challenge due to its detrimental effects on kidney function. Clopidogrel (Clop), an antiplatelet drug known for its ability to prevent blood clots by inhibiting platelet aggregation, also has potential effects on oxidative stress and cell death. This study investigates Clop’s protective role against Genta-induced nephrotoxicity, emphasizing the importance of the coagulation cascade. The 32 adult male albino rats were randomly assigned to four groups of eight (*n* = 8). The first group received only the vehicle. Genta was injected intraperitoneally at 100 mg/kg/day for 8 days in the second group. Groups 3 and 4 received oral Clop at 10 and 20 mg/kg/day for 1 week before Genta delivery and throughout the experiment. Renal tissue showed renal function tests, oxidative stress, pro-inflammatory cytokines, apoptotic markers, coagulation profile, and fibrin expression. Clop improved Genta-induced kidney function and histopathology. Clop substantially reduced pro-inflammatory cytokines, oxidative stress indicators, pro-apoptotic proteins, and fibrin protein. Clop also significantly boosted renal tissue anti-inflammatory and anti-apoptotic protein expression. Genta-induced nephrotoxicity involves oxidative stress, apoptosis, and coagulation system activation, according to studies. This study underscores that Genta-induced nephrotoxicity is associated with oxidative stress, apoptosis, and activation of the coagulation system. Clop’s protective effects on nephrons are attributed to its anticoagulant, antioxidant, anti-inflammatory, and anti-apoptotic properties, presenting it as a promising therapeutic strategy against Genta-induced kidney damage.

## Introduction

Nephrotoxicity induced by gentamicin (Genta), a widely used antibiotic belonging to the aminoglycoside class, represents a significant clinical concern due to its adverse effects on kidney function (Campbell et al. 2023). Genta, while effective in combating severe bacterial infections, can cause damage to renal structures, leading to acute renal failure (ARF) (Elsisi et al. 2021; Zarei and Elyasi 2022). While Genta-induced nephrotoxicity typically exhibits dose-dependent characteristics, multiple variables may augment the risk of such nephrotoxicity. These include heightened dosages, prolonged administration, pre-existing renal conditions, advanced age, and concurrent usage of other nephrotoxic agents (Perazella and Rosner 2022). Therefore, understanding the intricacies of gentamicin-induced nephrotoxicity remains crucial in improving patient care and developing strategies to mitigate the potential adverse effects on renal function.

The pathophysiology of Genta-induced nephrotoxicity involves a multifaceted process that affects the kidney at a cellular and molecular level (Sanchez-Gonzalez et al. 2011; Basile et al. 2012). The precise mechanisms behind this toxicity still need to be comprehensively understood (Huang et al. 2020). Genta accrues within the renal tubular cells, disrupting cellular functions and impairing the kidneys’ ability to filter waste products and maintain electrolyte balance (Lopez-Novoa et al. 2011; Pletz et al. 2018). It instigates the production of free radicles and mitochondrial dysfunction, culminating in cellular injury and apoptosis (Alsharidah et al. 2021; Yahyazadeh et al. 2021; Zarei and Elyasi 2022). Additionally, it disrupts the delicate balance of ions and transport mechanisms within the kidney, affecting its overall function.

The coagulation system, which involves complex processes leading to blood clot formation, plays a role beyond hemostasis (Palta et al. 2014). It is increasingly recognized for its involvement in various pathological conditions, including kidney injury. In the context of Genta-induced nephrotoxicity, researchers have suggested that the commencement of the coagulation cascade culminating in microvascular thrombotic occurrences within the kidney may precipitate endothelial injury in renal blood vessels and contribute to the damage seen in renal tissue (Verma and Molitoris 2015; Madhusudhan et al. 2016; Krishnan et al. 2021). The formation of small clots within the renal microvasculature can compromise blood flow, contributing to tissue damage and impaired kidney function (Sutton et al. 2002). Moreover, inflammatory pathways activated by Genta-induced injury might lead to the simultaneous activation of coagulation pathways. This crosstalk between inflammation and coagulation can further exacerbate kidney injury (Esmon 2008; Suárez-Álvarez et al. 2016). Therefore, understanding the interaction between coagulation pathways and kidney injury in the context of Genta-toxicity is an area of ongoing research.

While studies have indicated the involvement of the coagulation cascade in nephrotoxicity, the precise mechanisms and significance of Genta-induced kidney damage are still being explored. Recognizing the contribution of the coagulation system to kidney injury due to Genta administration is crucial for developing potential therapeutic approaches. These involve targeting coagulation pathways to mitigate thrombotic events and reduce the severity of kidney injury associated with using Genta.

Clopidogrel (Clop), an antiplatelet medication, primarily works by inhibiting platelet aggregation, reducing the risk of blood clots, and preventing cardiovascular events like heart attacks and strokes (Jiang et al. 2015). While the main mechanism of Clop is to inhibit platelet aggregation and prevent blood clot formation, some studies have suggested additional effects that might influence oxidative stress and cell death processes (Yip et al. 2012). Numerous preclinical studies suggest that Clop can reduce lipid peroxidation, decreasing the malondialdehyde (MDA) level, which is considered a recognized marker for the detrimental effects of oxidative stress. This effect was consistent across various animal models (Kanko et al. 2005; Hu et al. 2011; Hadi et al. 2013). Clop decreased ROS toxicity and prevented a decline in GSH, thereby enhancing the body’s natural antioxidant systems.

Additionally, studies have highlighted Clop’s safeguarding the kidneys from ischemia-reperfusion injury in murine models, specifically in preventing apoptotic cell death in kidney cells (Zhu et al. 2011). The safeguarding effect of Clop is also linked to its binding with the adenosine diphosphate (ADP)-binding purinergic receptor on platelets P2Y12.

P2Y12, a G-protein-coupled receptor, is predominantly expressed on the surface of platelets, where its activation initiates platelet aggregation (O’Connor et al. 2011). Excessive platelet activation has been implicated in the pathogenesis of myocardial infarction and stroke (Yeung et al. 2018). Recent research has broadened the scope of P2Y12 expression, demonstrating its presence in a diverse range of cell types. Notably, P2Y12 is expressed in immune cells such as monocytes (Micklewright et al. 2018), microglial cells (Moore et al. 2015), osteoblasts and osteoclasts (Mediero et al. 2016), tumor-associated macrophages (TAMs) (Kloss et al. 2019), as well as hepatic macrophages in the context of liver cirrhosis and hepatocellular carcinoma (Pavlović et al. 2020). Additionally, the expression of the P2Y2 receptor (P2Y2R) has been shown to be upregulated in polycystic kidney disease where it plays a role in promoting cyst formation and growth. Suppression of P2Y2R expression has been shown to reduce cyst growth and enhance renal function (Kraus et al. 2016). Activation of P2Y2R in these cells is linked to a diverse range of cellular activities, such as the release of cytokines, production of reactive oxygen species (ROS), induction of apoptosis, chloride secretion, cell proliferation, and changes in vascular structure (Arulkumaran et al. 2013; Solini et al. 2015).

Therefore, inhibiting the action of this receptor by Clop results in the suppression of aggregation and activation of platelets (Evangelista et al. 2005) and the suppression of free radical generation (Kanko et al. 2005). Consequently, this research aimed to assess how Clop might offer protection against nephrotoxicity caused by Genta, emphasizing the importance of addressing the activation of the coagulation cascade to mitigate Genta-induced nephrotoxicity.

## Materials and methods

### Kits and reagents

Genta was procured from Schering-Plough Egypt for the pharmaceutical industry. Clop was obtained as a gift from Sanofi Aventis Pharma for pharmaceutical products in Egypt. Renal function tests were conducted using the Creatinine assay kit (CAT# BK-472525D) and blood urea nitrogen (BUN) assay kit (CAT# BK-443350D) obtained from Diamond Diagnostics Company in Cairo, Egypt. Additionally, a kit for measuring gamma-glutamyl transferase (GGT) activity (CAT# 12023) was sourced from HUMAN Biochemical and Diagnostic Company based in Germany for the assessment of renal function. Prothrombin time (PT) kit (CAT# 210-08-010-00) and activated partial thromboplastin time (aPTT) kit (CAT# 210-09-050-00) were obtained from LABiTec GmbH Company, Germany, for the determination of rat’s coagulation profile. Additionally, a diagnostic kit for the detection of malondialdehyde (MDA) activity (CAT# E-BC-K025-S) and reduced glutathione (GSH) content (CAT# E-BC-K030-S), as well as nitrate/nitrite (NOx) production (CAT# E-BC-K135-M) in renal tissue, was acquired from Elabscience (TX, USA). Rat Elisa kits for assessing cystatin-c (Cyst-C) (CAT# CSB-E08385r) and Interleukin-10 (IL-10) (CAT# CSB-E04595r) were procured from CUSABIO in College Park, USA. For interleukin-6 (IL-6) (CAT# E-EL-R0015) and interleukin-1Beta (IL-1β) (CAT# E-EL-R0012), Rat Elisa kits were obtained from Elabscience in TX, USA. Mouse monoclonal anti-BAX (CAT# sc-20067), anti-Bcl2 (CAT# sc-7382), anti-NF-kβ (CAT# sc-514451), and anti-fibrin (CAT# sc-271035) were acquired from Santa Cruz Biotechnology Co. in CA, USA. Additionally, rabbit monoclonal anti-caspase-3 (CAT# 9662) was acquired from Cell Signaling Technology in BOS, USA. All other solvents and chemicals were of high analytical purity.

### Animals

For this study, adult male albino rats, weighing an average of 220 ± 30 g, were sourced from the Modern Veterinary Facilities for Laboratory Animals in Cairo, Egypt. Rats were fostered in conventional housing conditions with a temperature maintained at 25 °C ± 0.5 and a relative humidity of 55% ± 1 and a 12-h light/dark cycle before undergoing laboratory experiments. Throughout the study, the rats had unrestricted access to standard forage and water *ad libitum*. A day prior to concluding the experiment, individual metabolic cages were used to collect 24-h urine and measure urine volume. All animal care procedures adhered to regulatory guidelines and followed the principles outlined in the European Community (86/609/EEC Edition 8). The ethical aspects of animal care were endorsed by the Ethical Committee of Scientific Research at Nahda University, Beni-Sueif, Egypt, with the IACUC permit no. (NUB-019-023).

### Experimental design

Thirty-two male albino rats were divided into four groups, each consisting of 8 rats. Group 1, the normal control, received only the vehicle (saline in 1% v/v tween 80). Group 2, the Genta-nephrotoxic group, was injected with 100 mg/kg/day/i.p. of Genta for 8 consecutive days (Kasap et al. 2013). Groups 3 and 4, Genta + Clop 10 mg/kg and Genta + Clop 20 mg/kg, respectively, were treated with freshly prepared Clop solution in 1% v/v tween 80. Clop was administered orally at 10 mg/kg/day (Konosic et al. 2019) or 20 mg/kg/day (Khalaf et al. 2018), respectively, for 7 days before Genta administration persisted for the duration of the experiment.

### Urine, blood, and tissue sampling

Upon completing the experiment, every rat was raised individually in metabolic cages for 24 h to facilitate the compilation of urine. Urinary samples were utilized to evaluate creatinine clearance (Cr Cl) and gamma-glutamyl transferase (GGT) enzymatic activity. For blood sampling, the retro-orbital sinus was accessed under light ether anesthesia. A fraction of the blood was drawn into citrate tubes, followed by centrifugation, and the resulting supernatant was utilized for assessing prothrombin time (PT), concentration (PC), and activated partial thromboplastin time (aPTT).

Another blood portion was collected in EDTA tubes to determine the differential white blood cells (WBCs) and platelet count. The third portion was drawn into non-heparinized tubes and centrifuged at 3000 rpm for 15 min to isolate the serum. The serum was kept at a temperature of −20 °C for subsequent analysis of serum Cr, Cyst-C, and BUN concentration.

Following the experimental procedures, all animals underwent euthanasia through carbon dioxide inhalation. The kidneys were carefully uncapsulated, rinsed gently with saline, and their weights were recorded. The right kidney was promptly dissected, immersed in Davidson’s solution, and embedded in paraffin blocks for subsequent histological and immunofluorescence examinations. Simultaneously, the left kidney underwent homogenization and centrifugation, and the resulting supernatant was utilized to assess renal GSH and MDA, as well as NOx production.

A distinct segment of each kidney underwent homogenization in a pH 7.4 phosphate-buffered saline solution, followed by centrifugation under chilled condition at 4000 rpm. The supernatants were subsequently portioned into aliquots and preserved at −20 °C for subsequent estimation of additional biochemical parameters. Lastly, a portion of the renal tissue was preserved in a solution containing a protease inhibitor and subsequently frozen at −80 °C in preparation for eventual western blot analysis.

### Assessment of serum and urine renal function biomarkers

Commercially available diagnostic kits were employed to measure serum and urine creatinine, as well as BUN. The procedures strictly adhered to the manufacturer’s instructions (Richard et al. 1974; Charles and Crouch 1977), ensuring precision and consistency in the measurements. Creatinine clearance was calculated using a previously defined formula (Adikay and Koganti 2010) adding an extra layer of accuracy to the assessments.

$$Creatinine\;clearance= \frac{urine\;creatinine}{serum\;creatinine}\;\times\;\frac{urine\;volume\;(ml)}{1440}$$

Cystatin-C levels in serum and GGT enzymatic activity in urine were determined in adherence to the manufacturer’s directives (Lee et al. 2003).

### Assessment of differential WBCs and platelet count

For hematological analysis, an automated analyzer developed by ABX Micros from HORIBA Medical in France was employed. This advanced system counts and sizes blood cells, providing detailed insights into various parameters. The measured parameters encompass total white blood cell count (WBC), lymphocyte % (Lymp), mononuclear cell % (Mon), granulocyte % (Gr), and platelet count.

### Assessment of coagulation profile (PT and aPTT)

Prothrombin time (PT) and activated partial thromboplastin time (aPTT) were determined using the methodology detailed by Dacie and Lewis (2001). The assessments were carried out with a Coa-DATA automated analyzer, strictly following the manufacturer’s instructions.

### Assessment of renal oxidative stress parameters

The kidney homogeneity was meticulously examined through biochemical analysis to ascertain levels of MDA, glutathione GSH, and nitric oxide NOx production. This involved the utilization of colorimetric kits, with each step meticulously executed with reference to the manufacturers’ precise instructions.

### Assessment of renal IL-10, IL-1β, and IL-6 expression

In adherence to the guidelines stipulated by the manufacturers of ELISA kits acquired from CUSABIO (College Park, USA) and Elabscience (TX, USA), the expression levels of interleukin-10 (IL-10), interleukin-1b (IL-1b), and interleukin-6 (IL-6) in renal tissue samples were assessed. These assays were carried out in duplicate to ensure the accuracy and reliability of the results. Following the completion of the assays, absorbance readings were obtained using a sophisticated microplate reader, specifically the ELx 800TM model manufactured by BioTek, USA. This meticulous approach adhered to established protocols and quality control measures to maintain the integrity of the experimental data and facilitate comprehensive analysis of cytokine expression patterns in the renal tissue samples

### Western blot analysis

This technique was meticulously implemented, following a previously established protocol, to evaluate protein concentrations of NF-kB and caspase-3 in kidney tissue (Ewees et al. 2021). Protein immobilization was accomplished by transferring the samples loaded onto SDS-PAGE onto a Bio-Rad nitrocellulose membrane. The blocking step was conducted using a TBS-T blocking solution, which included 5% skim milk, and the membrane underwent incubation for a duration of 1 h at room temperature. During this time, non-specific binding sites were blocked, enhancing the specificity of subsequent antibody binding. The primary antibody was incubated overnight at 4 °C, ensuring thorough and specific binding of antibodies to their respective targets. After the primary antibody incubation, the membranes underwent careful rinsing in TBS-T to remove unbound antibodies. Subsequently, the membranes were subsequently exposed to secondary antibodies, which included anti-mouse, anti-rabbit, or anti-mouse HRP-coupled AP-coupled antibodies. This secondary antibody incubation lasted for 1 h, facilitating the detection of primary antibody-bound proteins. For visualization, a DAB (3,3′-Diaminobenzidine) detection kit (Chongqing et al., # BWR1069) or BCIP/NBT was employed.

### Assessment of renal histopathology

After the tissue sections were fixed, they were encased with paraffin wax and chopped into segments with a thickness of 4 µm. After staining with hematoxylin and eosin (H&E) was subsequently performed on these sections. In consequence, the stained sections were assessed for histopathological changes using Olympus light microscopy at a magnification of ×200, accompanied by an Olympus digital camera for documentation.

### Immunohistochemistry examination

Super-frosted paraffin tissue samples underwent a dual de-paraffinization process, spending 15 min each time immersed in 100% xylene post a 20-min stint in a 58 °C oven. A rehydration sequence ensued, involving a gradient approach with 100% ethanol for 5 min (twice), followed by a 5-min progression through ethanol concentrations of 90%, 70%, 50%, and 30%. Subsequently, the tissues experienced a 5-min rinse in distilled water before incubation in citrate buffer (pH 6.0) for antigen retrieval. To thwart any endogenous peroxidase activity, slides were treated with 3% H2O2 in methanol for 10 min. Nonspecific binding was preemptively blocked for 10–15 min using 10% normal goat serum. Subsequently, the slides encountered a 20-min interaction with the secondary antibody, horseradish peroxide (HRP)-polymer anti-mouse IgG, at room temperature, post an overnight exposure at 4 °C to the primary antibodies, anti-BAX and anti-Bcl2 mouse monoclonal antibodies. Hematoxylin served as the counteractive force against peroxidase activity, while color development was entrusted to diaminobenzidine (DAB). The evaluation of immunostaining took place under the watchful eye of the LEICA DM 2500 light microscope in New York, USA. Results interpretation involved a holistic consideration of both staining intensity and the percentage of positive cells. Reactivity was subsequently categorized on a scale ranging from negative (0) to weak (+), moderate (++), and intense (+++).

### Immunofluorescence examination

The immunofluorescence staining process adhered to previously established procedures (Abdel-Bakky et al. 2011). Sections underwent de-paraffinization in xylene and graduated rehydration through ethanol concentrations. The antigen retrieval process was executed by submerging the samples in a solution of 0.01 M sodium citrate buffer with a pH of 6 for a duration of 20 min. Following chilling, sections were rinsed with PBST (7.4 pH phosphate buffer saline containing 0.05% tween 20 in), fixed with p-formaldehyde (3.7%) for 10 min, and subsequently blocked at room temperature for 1 h using blocking buffer (PBS containing 1% BSA and 10% horse serum). After that, sections were incubated with primary antibodies (anti-fibrin mouse monoclonal antibody) overnight at 4 °C. After washing, tissue antigens were revealed through a 30-min interaction with goat anti-mouse Cy3 secondary antibodies. Counterstaining involved the application of 4,6-diamidino-2-phenylindole (DAPI), followed by a 30-min wash with PBST. Ultimately, the tissue sections were gently mounted using Fluoromount G and unveiled by fluorescence microscopy using Leica DM5000 B (Leica, Germany). The average fluorescence intensity from 3 to 5 microscopic fields per tissue section was quantified using ImageJ/NIH software. Subsequently, the obtained values were normalized to DAPI intensity for accurate comparison.

### Statistical analysis

Data analysis was conducted using the Social Science Statistical Package (SPSS) software, version 22.0. The results are reported as mean values with standard deviation error (SD). Statistical comparisons were made using one-way analysis of variance (ANOVA), followed by the least significant difference post-hoc test for multiple comparisons. Significance was established at *p* < 0.05.

## Results

### Effect of Clop on animal weight and relative kidney weight in rats with Genta-nephrotoxicity

Rats under normal conditions showed a consistent rise in body weight throughout the experiment, reaching an average of 34.38 ± 1.99 g by the end. Conversely, those subjected to Genta injections experienced a notable decline in weight, amounting to a significant decrease of 129.08% compared to normal rats. However, rats treated with varying doses of Clop (10 and 20 mg/kg) exhibited a remarkable surge in weight, with increases of approximately 2.38 and 3.75 folds, respectively, as opposed to the Genta-nephrotoxic group (Table 1).

**Table 1:** Effect of Clop on animal weight and relative kidney weight in rats with Genta-nephrotoxicity

	Initial weight (g)	Final weight (g)	∆Weight (g)	Kidney weight (g)	Relative kidney weight
Normal control	193.00 ± 10.94	228.75 ± 13.82	34.38 ± 5.63	0.79 ± 0.11	0.34 ± 0.03
Genta-nephrotoxicity	221.11 ± 15.54	211.01 ± 13.51	−10.06 ± 3.24^a^	1.11 ± 0.04	0.51 ± 0.03^a^
Genta + Clop 10 mg/kg	204.38 ± 20.94	228.13 ± 22.85	23.75 ± 3.53^ab^	0.87 ± 0.16	0.38 ± 0.048^b^
Genta + Clop 20 mg/kg	207.50 ± 14.39	245.01 ± 19.64	37.50 ± 12.25^abc^	0.93 ± 0.07	0.38 ± 0.01^b^

- Each value represents the mean of 6–8 experiments ± SD

- Statistical analysis was performed using one-way ANOVA followed by post-hoc test multiple comparisons test where

^a^Significantly different from the normal control group value at *p* < 0.05

^b^Significantly different from Genta-nephrotoxicity group value at *p* < 0.05

^c^Significantly different from Genta + Clop 10 mg/kg group value at *p* < 0.05

Additionally, the relative kidney weight of control rats remained stable at around 0.34 ± 0.011 g. In contrast, rats administered with Genta displayed a considerable rise in relative kidney weight, reaching approximately 1.46 folds compared to the control group. Conversely, rats treated with Clop at both low and high doses (10 and 20 mg/kg) demonstrated a significant decrease in relative kidney weight, plummeting to approximately 0.76 and 0.77 folds compared to the Genta-nephrotoxic group (Table 1).

### Effect of Clop on serum kidney function tests in rats with Genta-nephrotoxicity

**Fig. 1:**
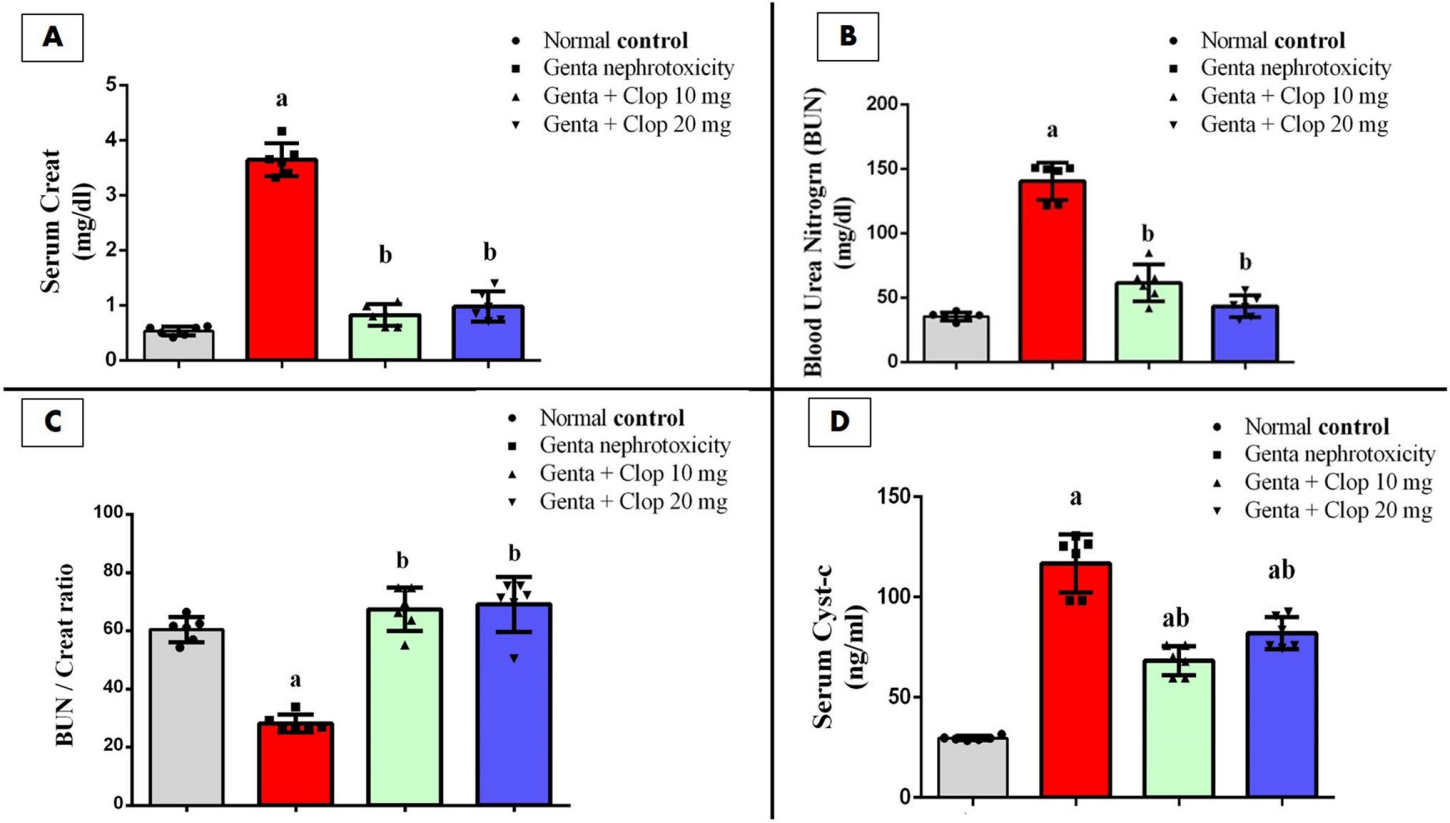
Effect of clopidogrel (Clop) on serum kidney function tests in rats with Genta-nephrotoxicity where** A** serum Cr, **B** BUN, **C** BUN/Cr ratio, and** D** serum Cyst-c. Data are displayed as mean±SD (*n* = 6–8). The statistical analysis involved conducting a one-way analysis of variance (ANOVA), which was then followed by a post-hoc multiple comparisons test where (a) the significance level for a difference from the normal control group value at *p* < 0.05; (b) The significance level for a difference from Genta-nephrotoxicity group value at *p* < 0.05

In the preceding study, Genta administration led to a notable deterioration in kidney function, as demonstrated by substantial rises in serum levels of Cr, Cyst-c, and BUN. These levels increased to around 720.72%, 350.33%, and 385.58%, respectively, compared to the normal control group (Fig. 1A, B, and D).

Conversely, administration of a low dose of Clop (10 mg/kg) to rats resulted in a substantial decrease in serum levels of Cr, Cyst-c, and BUN, dropping by roughly 27.5%, 64.59%, and 39.76%, respectively, compared to the groups exposed to Genta-induced nephrotoxicity. Furthermore, rats treated with higher doses of Clop (20 mg/kg) also exhibited substantial reductions in serum levels of Cr, Cyst-c, and BUN, declining by approximately 24.5%, 80.84%, and 28.57%, respectively, when compared to rats subjected to Genta-induced nephrotoxicity (Fig. 1A, B, and D).

We also observed that the BUN/Creatinine (BUC) ratio was significantly reduced to about 45.68% in the Genta-nephrotoxicity group compared to normal control rats. Meanwhile, both doses of Clop significantly increased the BUC ratio by 213.68% and 248.37%, respectively, as opposed to the Genta-nephrotoxic group (Fig. 1C).

### Effect of Clop on urine kidney function tests in rats with Genta-nephrotoxicity

**Fig. 2:**
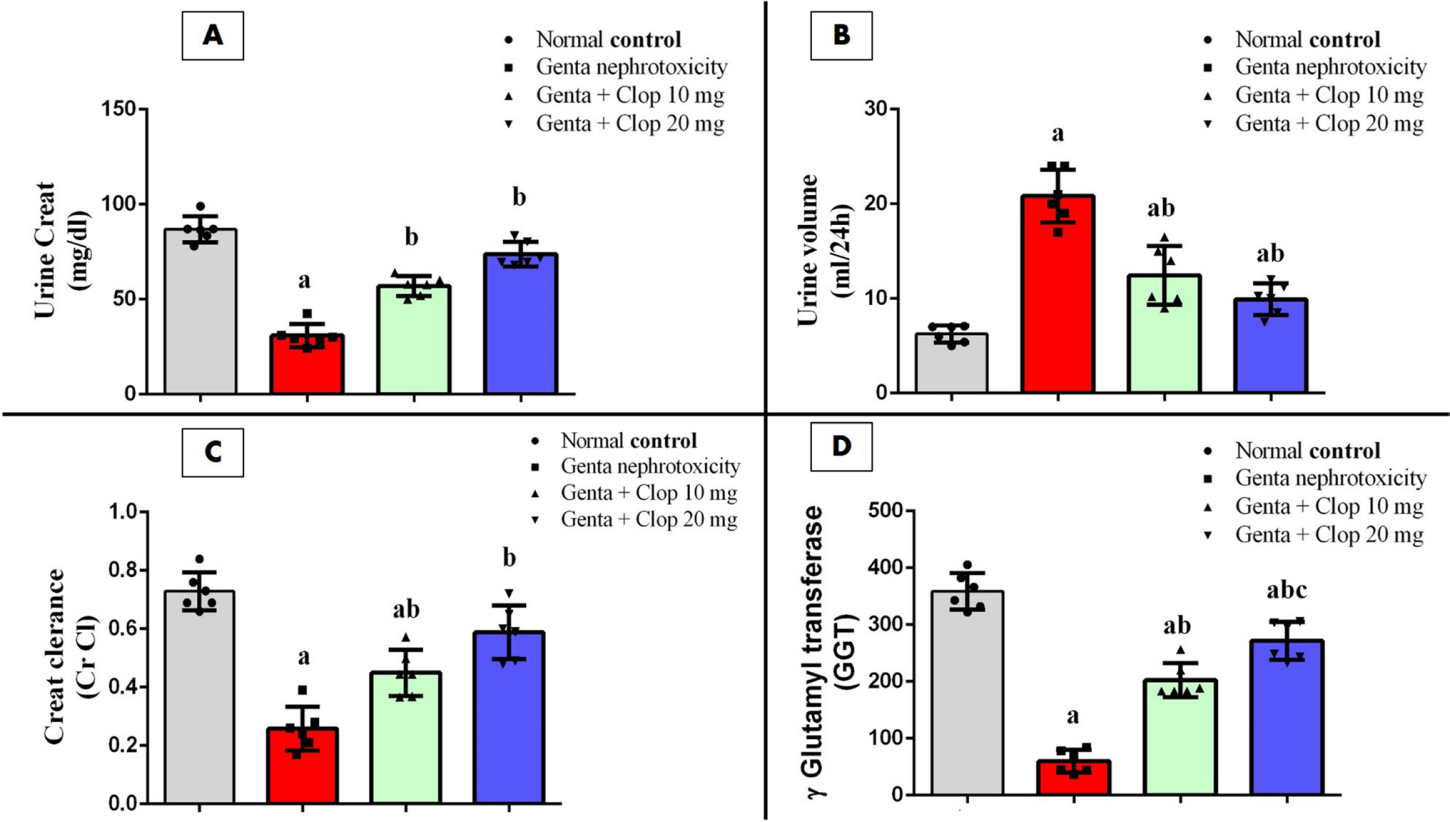
Effect of Clop on urine kidney function tests in rats with Genta-nephrotoxicity where **A** urine Cr, **B** urine volume, **C** Cr Cl, and **D** GGT. Data are displayed as mean±SD (*n* = 6–8). The statistical analysis involved conducting a one-way analysis of variance (ANOVA), which was then followed by a post-hoc multiple comparisons test where (a) the significance level for a difference from the normal control group value at *p* < 0.05; (b) the significance level for a difference from Genta-nephrotoxicity group value at *p* < 0.05

Healthy rats exhibited mean values of 81.07 ± 6.6 mg/dl for U. Cr, 5.81 ± 0.41 ml for urine volume, and 0.76 ± 0.065 ml/min for Cr Cl. Conversely, the injection of Genta generated a notable decline in urine Cr and Cr Cl levels, by approximately 37.87% and 32.89%, respectively, alongside a significant elevation in urine volume, by about 303.03%, as opposed to the normal control group (Fig. 2A–C).

Conversely, administering rats with both doses of Clop resulted in significant rises in urine Cr and Cr Cl levels. Specifically, in rats given Clop at 10 mg/kg, urine Cr and Cr Cl levels increased to about 149.31% and 160%, respectively, compared to the Genta-induced nephrotoxic group. Similarly, rats treated with Clop at 20 mg/kg showed significant increases, with urine Cr and Cr Cl levels reaching approximately 224.31% and 232%, respectively, compared to the Genta-induced nephrotoxic group (Fig. 2A, C). Furthermore, there was a significant reduction in urine volume following treatment with either low or high doses of Clop. Specifically, urine volume decreased to about 52.05% and 32.95%, respectively, compared to Genta-nephrotoxic rats (Fig. 2B).

We noted a considerable reduction in GGT levels in rat urine, dropping to approximately 14.55% following Genta administration compared to normal control rats. Conversely, treating rats with a low dose of Clop resulted in a significant increase in GGT levels, rising to about 354.58%. Furthermore, the high dose of Clop significantly elevated GGT levels to about 517.55% compared to the Genta-induced nephrotoxic group (Fig. 2D).

### Effect of Clop on differential WBCs and platelet count in rats with Genta-nephrotoxicity

In this study, rats injected with a single intraperitoneal dose of Genta exhibited a notable increase in total WBCs, lymphocytes, monocytes, and granulocytes, reaching approximately 1.49, 1.71, 1.58, and 1.95 folds higher, respectively, as opposed to normal control rats (Table 2). Conversely, treatment with Clop at 10 mg/kg significantly reduced WBCs, lymphocytes, monocytes, and granulocytes to about 69.70%, 71.98%, 55.37%, and 60%, respectively, compared to the Genta-induced nephrotoxic group. Furthermore, rats treated with Clop at 20 mg/kg displayed significant decreases in WBCs, lymphocytes, monocytes, and granulocytes to about 80.94%, 81.05%, 57.52%, and 66.67%, respectively, compared to rats with Genta-induced nephrotoxicity (Table 2).

**Table 2:** Effect of Clop on differential white blood cells in rats with Genta-nephrotoxicity

	WBCs (10^3^/mm^3^)	Lymph (10^3^/mm^3^)	Mon (10^3^/mm^3^)	Gran (10^3^/mm^3^)	Platelet count (10^3^/mm^3^)
Normal control	9.77 ± 1.91	7.22 ± 1.72	1.18 ± 0.32	0.23 ± 0.60	752.33 ± 33.99
Genta-nephrotoxicity	14.59 ± 2.51^a^	12.35 ± 2.64^a^	1.86 ± 0.34^a^	0.45 ± 0.11^a^	615.30 ± 79.62^a^
Genta + Clop 10 mg/kg	10.17 ± 3.01^b^	8.89 ± 2.55^b^	1.03 ± 0.36^b^	0.27 ± 0.07^b^	673.45 ± 84.07^b^
Genta + Clop 20 mg/kg	11.81 ± 2.77^ab^	10.01 ± 2.24^b^	1.07 ± 0.40^b^	0.30 ± 0.11^b^	733.50 ± 137.7^b^

Each value represents the mean of 6–8 experiments ± SD

Statistical analysis was performed using one-way ANOVA followed by a post-hoc multiple comparisons test where

^a^Significantly different from the normal control group value at *p* < 0.05

^b^Significantly different from Genta-nephrotoxicity group value at *p* < 0.05

In our observations, the *i.p* administration of Genta led to a considerable reduction in platelet counts, diminishing to about 81.79% compared to normal control rats. However, pretreating animals with Clop (at doses of 10 or 20 mg/kg) resulted in a significant elevation of platelet counts, increasing to approximately 1.10 and 1.20 times, respectively, compared to animals subjected to Genta-induced nephrotoxicity (Table 2).

### Effect of Clop on coagulation profile in rats with Genta-nephrotoxicity

As it was shown in Table 3, Genta-treated rats showed a marked increase in PT, aPTT, INR, and clotting time to about 1.6 folds, 1.53 folds, 1.46 folds, and 1.70 folds, respectively, compared to normal control rats. Conversely, there was a notable decrease in PC to approximately 43.83% was observed in rats injected with Genta as opposed to normal control rats (Table 3). On the contrary, Clop-treated rats demonstrated a considerable reduction in PT, aPTT, INR, and clotting time to about 58.35%, 67.65%, 62.5%, and 67.35%, respectively in low doses of Clop and about 58.29%, 71.38%, 63.23%, and 62.85%, respectively in high dose of Clop comparing to Genta-nephrotoxic group. Meanwhile, PC value significantly increased in rats treated with a low dose of Clop to about 2.59 folds and about 2.4 folds in rats treated with a high dose of Clop as compared to the Genta-nephrotoxic group (Table 3).

**Table 3:** Effect of Clop on blood coagulation profile; PT, PC, aPTT, and INR in rats with Genta-nephrotoxicity

	PT (sec.)	PC (U/ml)	INR	aPTT (sec.)	Clot. time (sec.)
Normal control	10.10 ± 0.43	121.24 ± 11.40	0.93 ± 0.03	19.63 ± 0.74	3.13 ± 0.99
Genta-nephrotoxicity	16.16 ± 1.80^a^	53.14 ± 9.65^a^	1.36 ± 0.12^a^	29.56 ± 5.53^a^	5.33 ± 0.79^a^
Genta + Clop 10 mg/kg	9.43 ± 1.82^b^	137.51 ± 29.81^b^	0.85 ± 0.14^b^	19.98 ± 2.93^b^	3.59 ± 0.92^b^
Genta + Clop 20 mg/kg	9.42 ± 1.30^b^	128.06 ± 44.54^b^	0.86 ± 0.10^b^	21.10 ± 3.21^b^	3.35 ± 1.33^b^

Each value represents the mean of 6–8 experiments ± SD

Statistical analysis was performed using one-way ANOVA followed by a post-hoc multiple comparisons test where

^a^Significantly different from the normal control group value at *p* < 0.05

^b^Significantly different from Genta-nephrotoxicity group value at *p* < 0.05

### Effect of Clop on renal oxidative stress in rats with Genta-nephrotoxicity

**Fig. 3:**
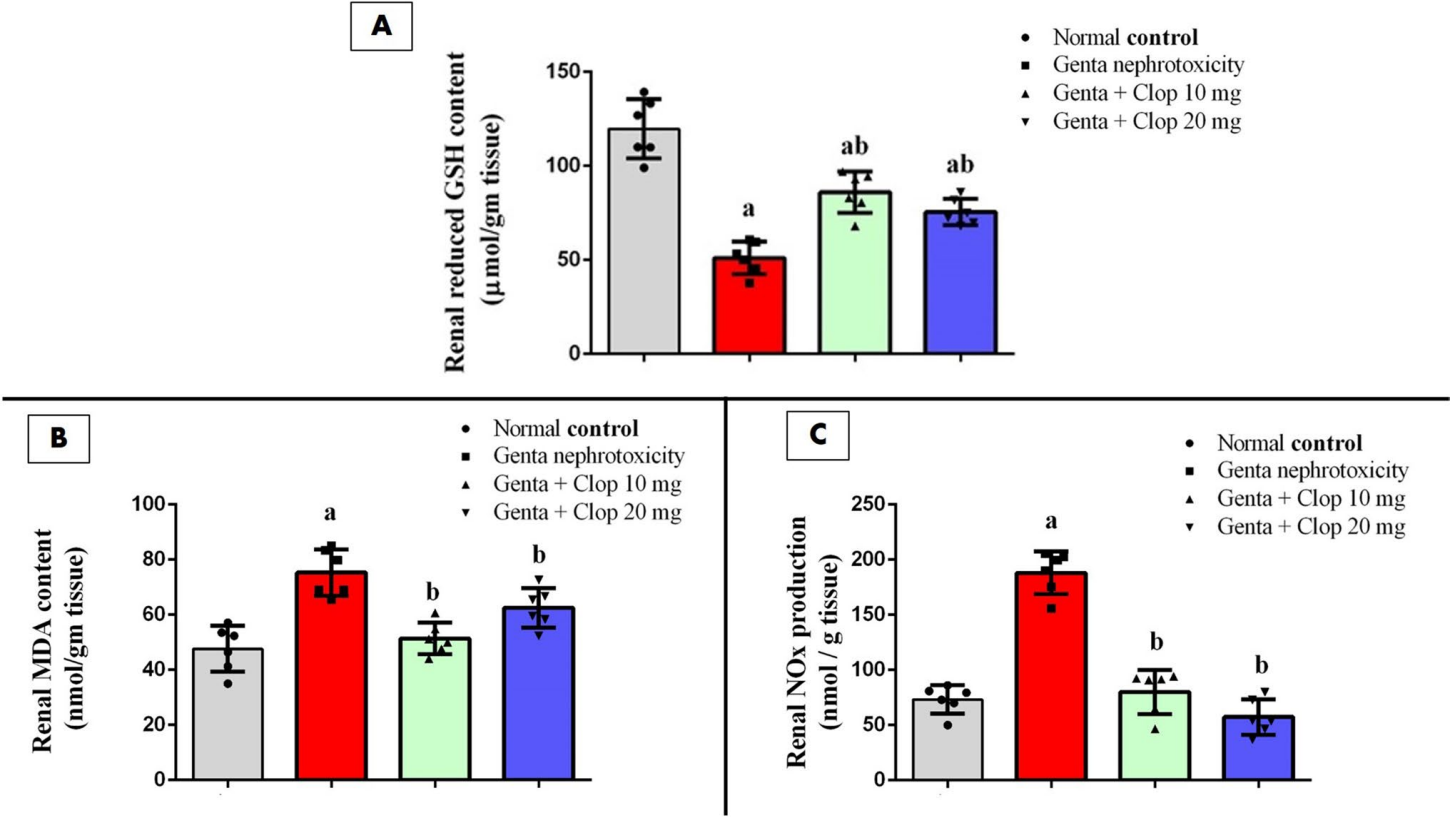
Effect of Clop on renal oxidative stress in rats with Genta-nephrotoxicity where **A** reduced GSH, **B** MDA content, and **C** NOx production. Data are displayed as mean±SD (*n* = 6–8). The statistical analysis involved conducting a one-way analysis of variance (ANOVA), which was then followed by a post-hoc multiple comparisons test where (a) the significance level for a difference from the normal control group value at *p* < 0.05; (b) the significance level for a difference from Genta-nephrotoxicity group value at *p* < 0.05

As detailed in Table 3, rats subjected to Genta treatment exhibited a notable increase in PT, aPTT, INR, and clotting time, rising to approximately 1.6-fold, 1.51-fold, 1.46-fold, and 1.70-fold, respectively, in comparison to normal control rats. Conversely, there was a significant decrease in PC, dropping to about 43.83%, in rats administered Genta as opposed to the normal control group (Fig. 3A–C).

In contrast, rats treated with Clop showed a significant reduction in PT, aPTT, INR, and clotting time to approximately 58.35%, 67.65%, 62.5%, and 67.35%, respectively, at low doses, and about 58.29%, 71.38%, 63.23%, and 62.85%, respectively, at high doses, compared to the Genta-induced nephrotoxic group. Additionally, PC values significantly increased in rats receiving a low dose of Clop to about 2.59-fold and in those receiving a high dose to about 2.4-fold compared to the Genta-induced nephrotoxic group (Fig. 3A–C).

### Effect of Clop on the expression of pro- and anti-inflammatory mediators in the kidney of rats with Genta-nephrotoxicity

**Fig. 4:**
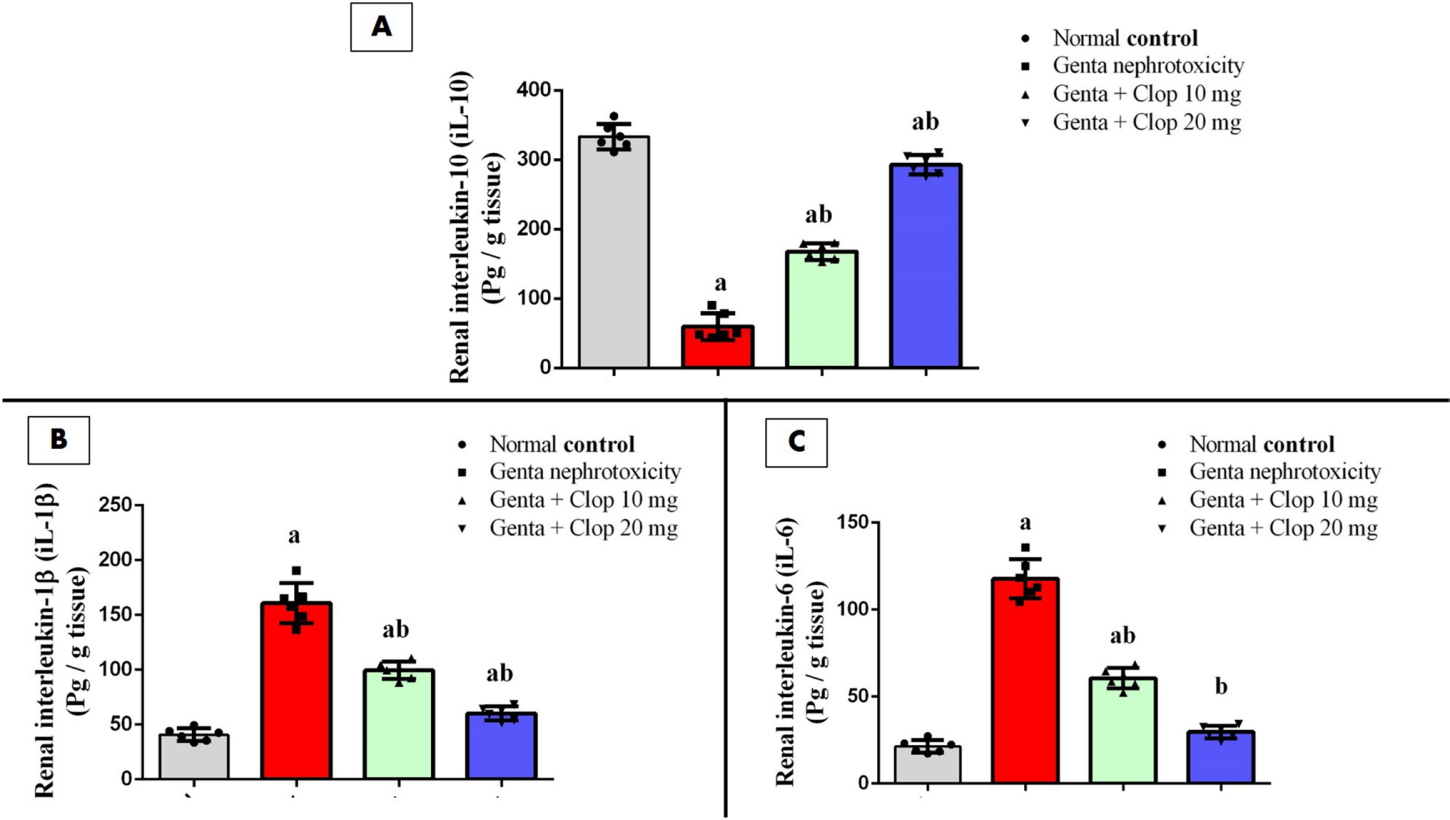
Effect of Clop on the expression of pro- and anti-inflammatory mediators in the kidney of rats with Genta-nephrotoxicity where **A** IL-10, **B** IL-1β, and **D** IL-6. Data are displayed as mean±SD (*n* = 6–8). The statistical analysis involved conducting a one-way analysis of variance (ANOVA), which was then followed by a post-hoc multiple comparisons test where (a) the significance level for a difference from the normal control group value at *p* < 0.05; (b) the significance level for a difference from Genta-nephrotoxicity group value at *p* < 0.05

In our investigation, the *i.p* administration of Genta was associated with a notable reduction in IL-10 protein expression, decreasing to approximately 0.42-fold compared to the normal control group. Conversely, treatment with either a low dose of Clop (10 mg/kg) or a high dose of Clop (20 mg/kg) resulted in a significant upregulation of IL-10 protein expression, increasing to about 4.07-fold and 7.36-fold, respectively, as opposed to the Genta-induced nephrotoxicity group (Fig. 4A).

Conversely, Genta administration precipitated a significant rise in the expression of IL-1β and IL-6 proteins, reaching approximately 4.24-fold and 5.7-fold, respectively, compared to the normal control group. In contrast, treatment of rats with either a low dose (10 mg/kg) or high dose (20 mg/kg) of Clop exhibited a significant decrease in IL-1β protein expression to approximately 61.19% and 35.90%, respectively, as well as a significant decrease in IL-6 to about 50.86% and 24.73%, respectively, compared to rats with Genta-induced nephrotoxicity (Fig. 4B, C).

Furthermore, the kidneys of normal control rats exhibited a basal expression of NF-kB protein density measuring 1.005 ± 0.003. Conversely, animals that received an injection of Genta showed a notable rise in NF-kB protein density expression, approximately 3.4 folds higher than that of normal control rats. Meanwhile, treatment with either low or high doses of Clop resulted in a significant reduction in NF-kB protein density expression, reaching approximately 66.41% and 37.98%, respectively, compared to the Genta-induced nephrotoxic group (Fig. 6A).

### Effect of Clop on the expression of apoptotic proteins in the kidney of rats with Genta-nephrotoxicity

**Fig. 5:**
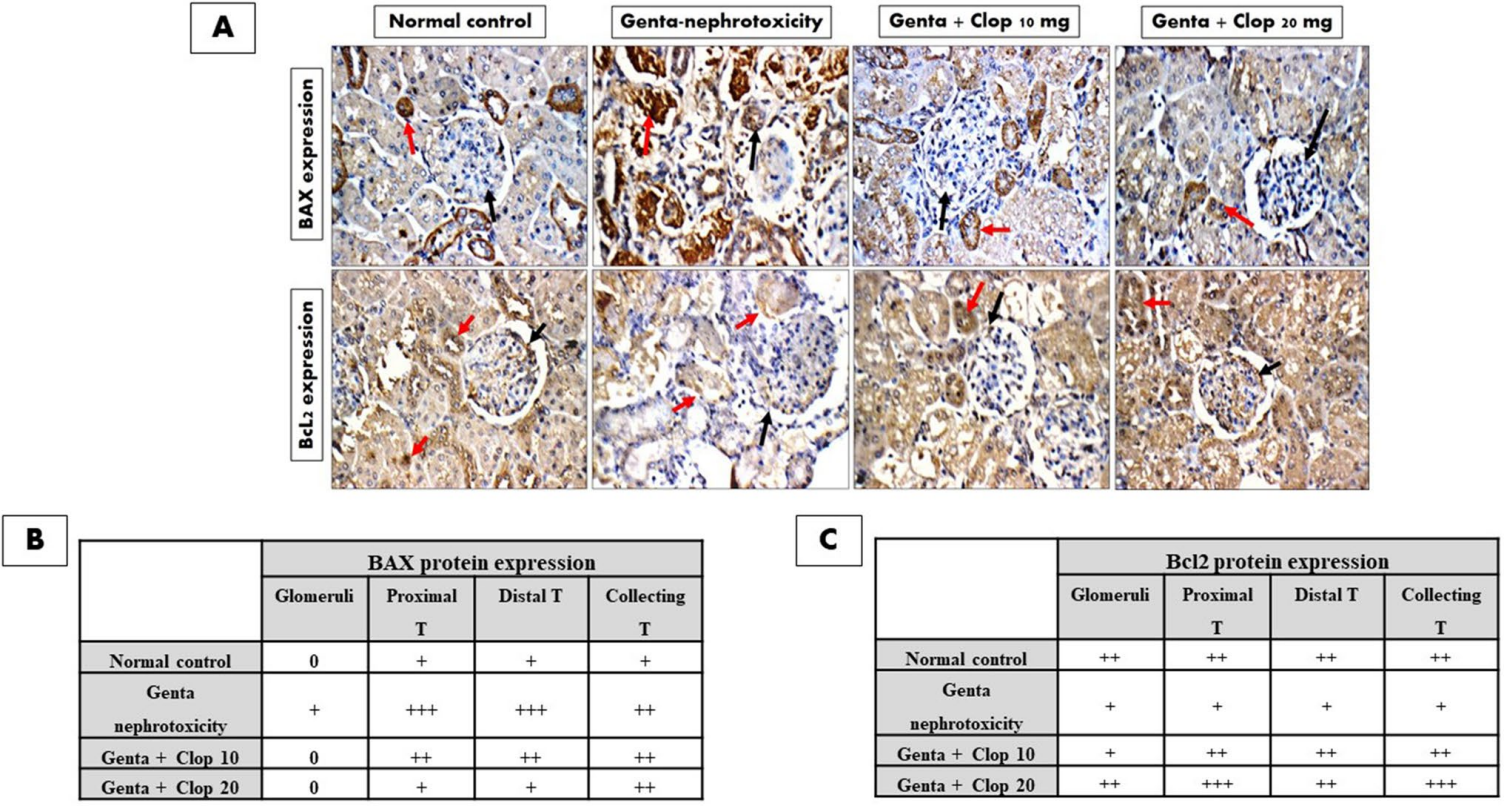
Effect of Clop on expression of BAX and Bcl2 proteins in the kidney of rats with Genta-nephrotoxicity where **A** expression of BAX and Bcl2, **B** analysis of BAX protein expression, and **C** analysis of Bcl2 protein expression

As depicted in Fig. 5, the kidney sections from normal control rats exhibited minimal cytoplasmic reactivity (0) for BAX protein in glomeruli and mild cytoplasmic reactivity (+) in proximal, distal, and collecting tubules. In contrast, the kidneys of rats in the Genta-induced nephrotoxicity group displayed marked cytoplasmic reactivity (+++) for BAX in proximal and distal tubules and moderate cytoplasmic reactivity (++) in collecting tubules (Fig. 5A, B). On the contrary, the kidneys of rats treated with a low dose of Clop (10 mg/kg) exhibited negative cytoplasmic reactivity (0) for BAX in glomeruli, moderate cytoplasmic reactivity (++) in proximal and distal tubules, and collecting tubules (Fig. 5A, B). Additionally, the kidney of rats treated with high doses of Clop (20 mg/kg) showed negative cytoplasmic reactivity (0) for BAX in glomeruli, weak cytoplasmic reactivity (+) in proximal and distal tubules, and moderate cytoplasmic reactivity (++) in collecting tubules (Fig. 5A, B).

Conversely, the kidney sections from normal control rats exhibited moderate cytoplasmic reactivity (++) for Bcl-2 in both glomeruli and tubules. In contrast, kidneys from animals treated with Genta showed weak cytoplasmic reactivity (+) for Bcl-2 in both glomeruli and tubules (Fig. 5A, C). Alternatively, animals receiving a low dose of Clop exhibited weak cytoplasmic reactivity (+) for Bcl-2 in glomeruli and collecting tubules, alongside moderate cytoplasmic reactivity (++) in proximal and distal tubules. Moreover, kidneys of animals treated with a high dose of Clop demonstrated moderate cytoplasmic reactivity (++) for Bcl-2 in glomeruli and marked cytoplasmic reactivity (+++) in tubules (Fig. 5A, C).

**Fig. 6:**
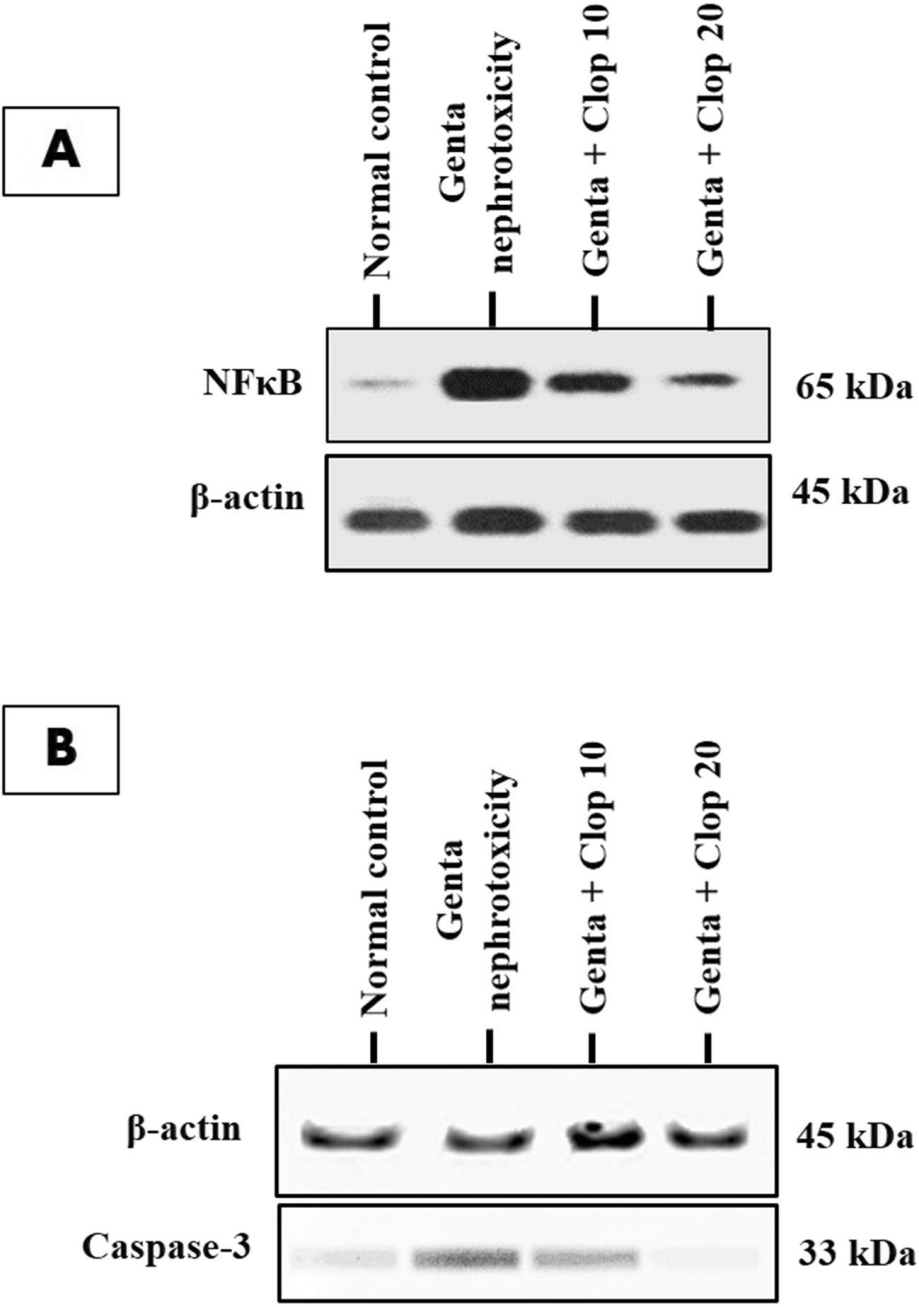
Effect of Clop on expression of NF-_K_β and Caspase-3 proteins in the kidney of rats with Genta-nephrotoxicity where **A** NF-_K_β and **B** Caspase-3

Moreover, normal control rats exhibited a slight expression of caspase-3 protein density measuring 1.339 ± 0.011. Conversely, intraperitoneal administration of Genta led to a significant increase in the expression of caspase-3 protein density, reaching 11.3 times compared to normal control rats. Meanwhile, rats treated with either Clop at 10 or 20 mg/kg displayed a significant decrease in the expression of caspase-3 proteins to approximately 48.77% and 34.58%, respectively, compared to the Genta-induced nephrotoxic group (Fig. 6B).

### Effect of Clop on the expression of fibrin protein in the kidney of rats with Genta-nephrotoxicity

**Fig. 7:**
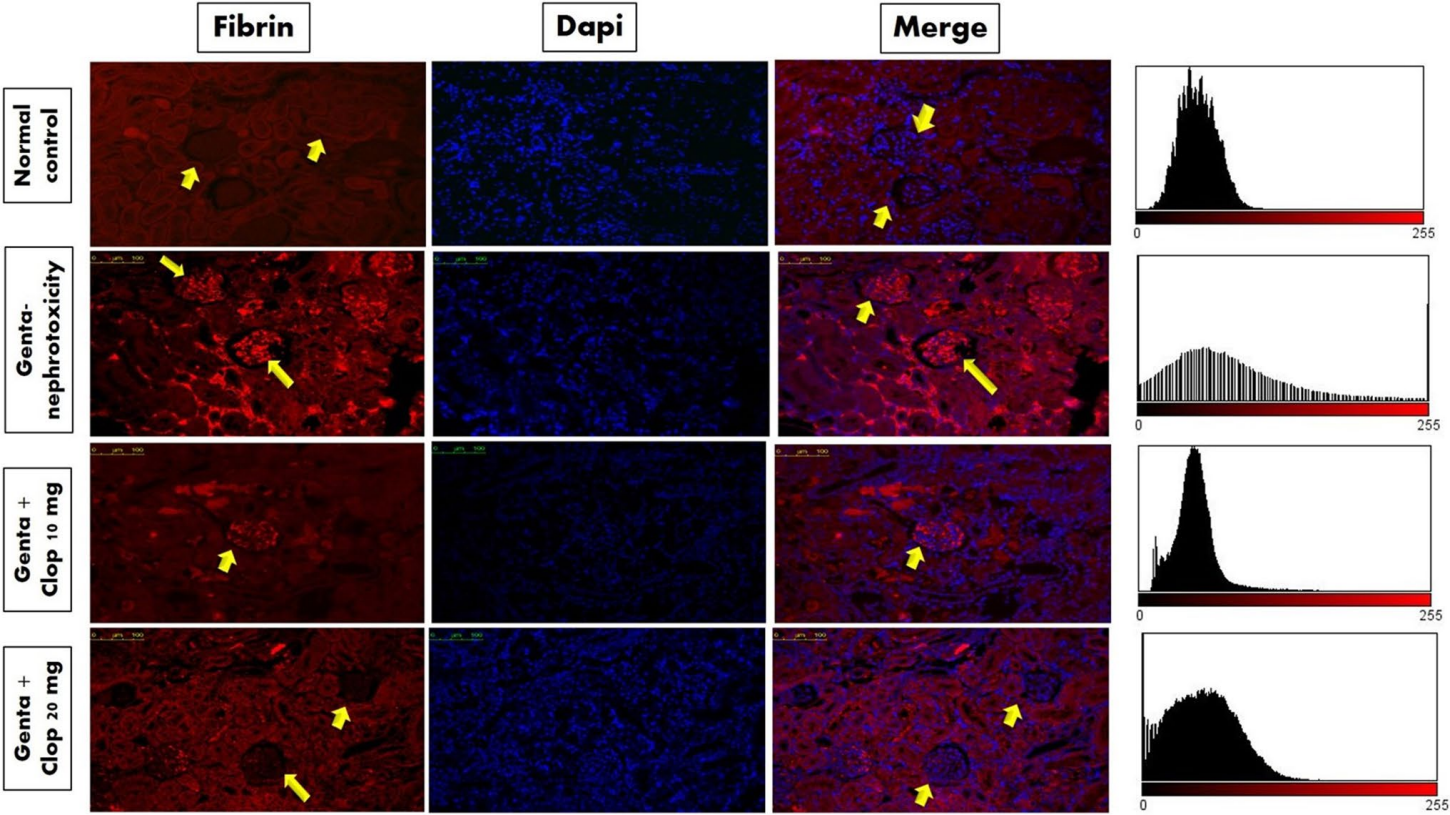
Effect of Clop on expression of fibrin protein in kidney of rats with Genta-nephrotoxicity

In normal rats, fibrin protein expression was not observed in renal tissue, either glomeruli or tubules. Meanwhile, rat kidneys treated with Genta 100 mg/kg *i.p.* showed high fibrin protein expression in renal tubular and glomerular cells as opposed to normal control. On the flip side, small and high doses of Clop markedly reduced the expression of fibrin protein in both renal glomeruli and tubule cells as opposed to the Genta-nephrotoxic group (Fig. 7).

### Effect of Clop on renal histological features in the kidney of rats with Genta-nephrotoxicity

**Fig. 8:**
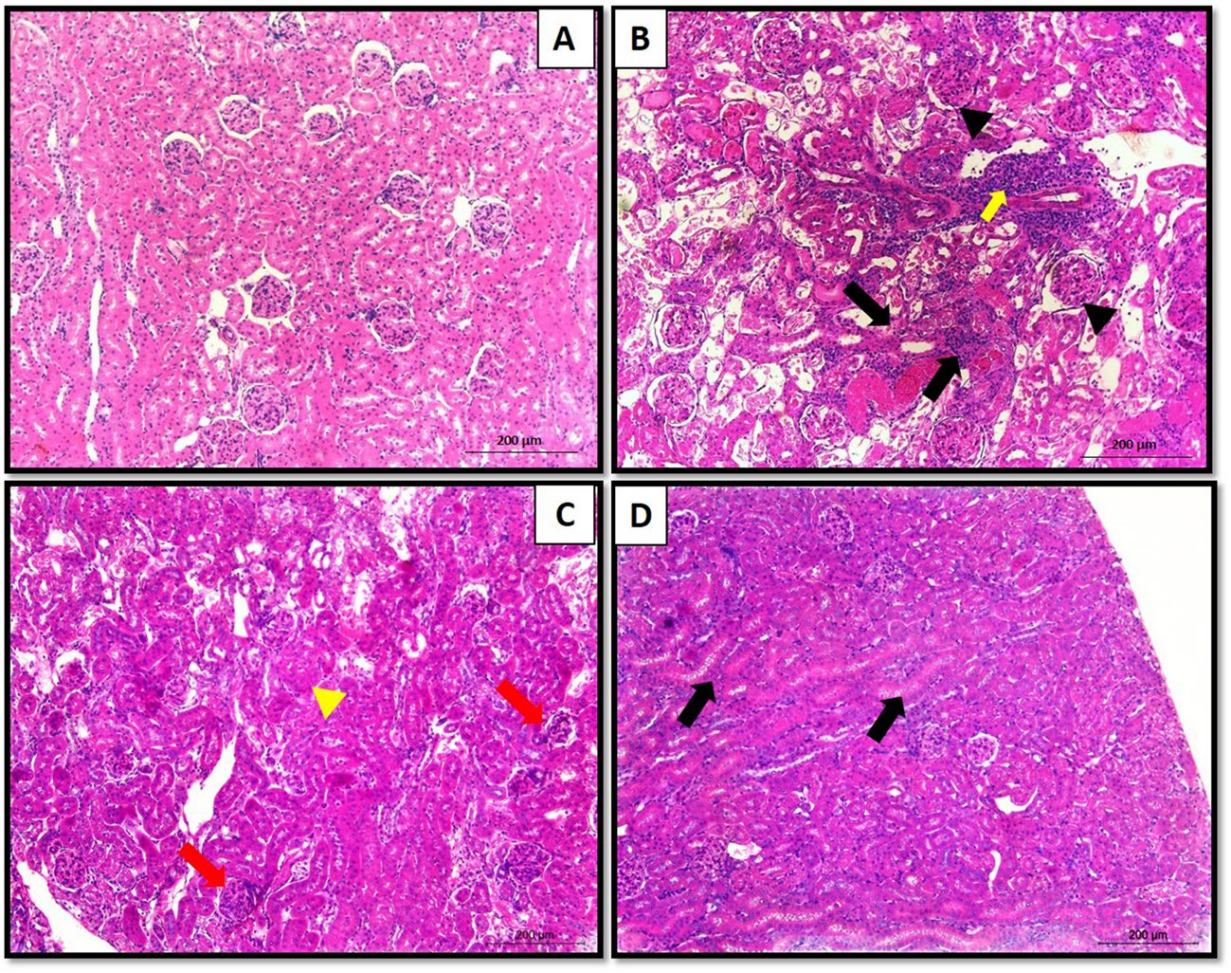
Effect of Clop on histological characters of kidney of rats with Genta-nephrotoxicity where **A** normal control group, **B** Genta-nephrotoxicity group, **C** Genta + Clop 10 mg/kg, and **D** Genta + Clop 20 mg/kg

In our observation, photomicrographs of kidney sections from normal control rats stained with (H&E) revealed a typical histological structure of renal glomeruli, alongside an intact Bowman’s capsule. The renal tubules displayed a relatively normal brush-bordered cuboidal lining epithelium (Fig. 8A). Conversely, upon histopathological examination of kidney sections obtained from rats injected with Genta, severe necrosis of renal lining epitheliums was evident, characterized by desquamation of most epithelial cells and pronounced degenerative changes (black arrow). Additionally, necrosis was observed in adjacent glomeruli (blackhead), accompanied by extensive leukocytic infiltration in the interstitial areas (yellow arrow) (Fig. 8B).

In contrast, animals pretreated with low doses of Clop exhibited moderate epithelial degeneration glomerulonephrosis (red arrow). Additionally, mild focal leukocytic infiltration was discernible in the interstitial area (yellow head) (Fig. 8C). Furthermore, high doses of Clop exhibited mild epithelial degeneration, with tubular lumens showing minimal lymphocytic infiltration (black arrow) (Fig. 8D).

## Discussion

The kidneys assume a pivotal role as primary targets susceptible to toxicity induced by chemicals owing to their integral involvement in the removal of harmful substances and their byproducts (Nassan et al. 2021). Numerous therapeutic agents have been documented for their tendency to cause nephrotoxicity during clinical usage (Perazella 2018). Genta, a frequently prescribed aminoglycoside antibiotic, is widely recognized as a significant contributor to drug-induced kidney damage (Randjelović et al. 2017). In this situation, the current study aimed to examine the potential nephroprotective effects of Clop in cases of kidney injury induced by Genta. This study represents the first to emphasize the role of Clop in alleviating nephrotoxicity caused by Genta.

In the present investigation, rats administered Genta displayed indications of kidney toxicity, as indicated by an increase in relative kidney weight, elevated serum Cr, BUN, Cyst-c, and urine GGT levels, and a reduction in Cr Cl. Furthermore, pronounced renal histological changes were observed, including necrosis of renal lining epitheliums, severe degenerative alterations, and necrosis of adjacent glomeruli. Additionally, extensive leukocytic infiltration in the interstitial areas was markedly evident. These results agree with previous studies that have shown similar results (Sharawy and Serrya 2020; Botros et al. 2022; Burgucu et al. 2022; Matouk et al. 2023; Nadeem et al. 2023).

Conversely, Clop showed significant enhancement in kidney functions, as manifested by a notable reduction in serum Cr, BUN, Cyst-c, and urine GGT levels, coupled with an enhancement in Cr Cl. These improvements were further supported by the positive changes observed in the histological examination. These findings align with earlier studies where the authors reported Clop’s capacity to enhance kidney function in diverse animal models (Tu et al. 2008; Hu et al. 2011; Wu et al. 2022).

Multiple investigations have confirmed that Genta elevates markers of oxidative stress while reducing markers of antioxidants, providing evidence that oxidative stress contributes to the renal toxicity caused by Genta (Al-Kuraishy et al. 2020; Abdelkader et al. 2022; Matouk et al. 2023; Saeedavi et al. 2023). Genta accumulates in the renal proximal convoluted tubules, leading to degeneration of the brush border membranes, generation of free radicals, reduction in antioxidant defenses, and resultant manifestations such as congestion in glomeruli, acute necrosis in tubules, and eventual failure of the kidneys (Abdel-Raheem et al. 2009; Balakumar et al. 2010).

Reactive oxygen species (ROS) are exceedingly reactive molecules that exert significant effects on membrane lipids, nucleic acids, and intracellular proteins, causing alterations in their functions and structures and resulting in cellular damage (Özcan et al. 2015). MDA, a byproduct of lipid peroxidation, is known to disrupt enzyme activities, increase cell membrane permeability, disrupt the balance of intracellular ions by impacting the exchange of ions across the membrane, and induce breaks and modifications in DNA structure (Erçin et al. 2019). Conversely, glutathione (GSH) assumes a crucial role in cellular maintenance. Thus, the reduction in renal GSH content may impede the body’s defense against the elevated ROS levels induced by Genta. Similarly, Genta administration leads to renal oxidative damage by depleting antioxidant defense enzymes.

Renal oxidative damage ensues from Genta’s induction of a deficiency in antioxidant defense enzymes. Additionally, Genta exerts nephrotoxicity by triggering the generation of cytokines that promote inflammation in the proximity of renal tissues (Qu et al. 2019). The crosstalk between ROS and inflammation becomes particularly significant in different toxicities and diseases. Persistent oxidative stress contributes to tissue damage, releases inflammatory mediators, and perpetuates a pro-inflammatory environment. Inflammatory responses are integral to the pathophysiology of acute renal failure (ARF), wherein the liberation of diverse cytokines into the damaged renal cells is pivotal for both the onset and advancement of ARF (Simmons et al. 2004; Ali et al. 2011).

The current investigation found that Genta markedly elevated IL-1β and IL-6 levels while reducing the expression of the anti-inflammatory cytokine IL-10 in the renal tissue of treated rats. These results suggest that inflammatory cytokines may be key players in the progression of Genta-induced proximal tubule dysfunction and nephrotoxicity. Interestingly, our findings contradict previous studies which reported a significant rise in pro-inflammatory cytokines IL-6 and IL-1β following Genta administration in renal tissue (Abdelkader et al. 2022; Bai et al. 2023; Nadeem et al. 2023). Additionally, our results confirmed the results observed by Samarghandian et al. (2015), who reported that injection of rats with Genta (100 mg/kg/ days) for 8 days caused a marked elevation in the expression of iL-6 and iL-1B as well as a reduction in the expression of anti-inflammatory cytokines Il-10 in renal tissue.

It has been documented that oxidative stress can activate various pro-inflammatory cytokines through the activation of NFκB (Sherif et al. 2015). NF-kB governs diverse cellular responses including innate immunity, inflammation, and apoptosis (Kumar et al. 2015). Its expression is evident in renal impairment and inflammatory reactions (Ansari et al. 2017). Hence, the induction of NF-kB triggered by ROS could potentially contribute to nephrotoxicity induced by Genta.

In our investigation, we noted a significant increase in NF-kB levels in the renal tissue of rats treated with Genta. These findings are consistent with a study by Nadeem et al. (2023), where it was reported that administration of Genta for 10 consecutive days led to a notable elevation in NF-kB levels in the renal tissue of Genta-treated rats.

One of the pivotal discoveries of this investigation is the anti-inflammatory effect of Clop against Genta-induced nephrotoxicity. We observed that Clop led to a marked decrease in the expression of pro-inflammatory cytokines, IL-1β and IL-6, coupled with an increase in the expression of IL-10 in renal tissue. These results were confirmed by other previous studies in which the potent anti-inflammatory effects of Clop were reported (Yip et al. 2012; Khalaf et al. 2017; Ishimatsu et al. 2020; Chen et al. 2022). A notable discovery in our investigation was the observed reduction in NF-kB expression in the renal tissue of rats injected with Genta upon treatment with Clop. Our findings align with those of Jia et al. (2019), who described that Clop mitigates dysfunction in vein endothelial cells induced by LPS by inhibiting the NF-kB signaling pathway.

The inflammatory response due to the overproduction of ROS was not limited to the increased release of cytokines but also affected blood cells and caused an increase in WBC counts, called leukocytosis. An extremely significant finding in our research is the marked elevation in WBCs and reduction in platelet count observed in Genta-treated rats. These findings are inconsistent with previous studies that confirmed the significant change in WBCs and platelet counts that appeared upon administration of Genta (Abhirama et al. 2018; Kondera et al. 2020; Aurori et al. 2023). On the contrary, pretreatment of animals with Clop caused a marked reduction in WBCs and caused renormalization to platelet counts.

Leukocytes, inflammatory cells, offer valuable insights into the detection of inflammatory diseases and the characterization of inflammation regarding its severity and type (Tvedten and Raskin 2012). Furthermore, emerging studies suggest that platelets are implicated in inflammation, infection, host response, and cancer.

Platelets express adhesion molecules and secrete them to facilitate their accumulation at damaged sites. These molecules promote platelet adhesion to leukocytes and granulocytes. Additionally, platelets release chemotactic immune modulators that attract neutrophils, monocytes, and lymphocytes. This interaction leads to the formation of platelet-granulocyte or platelet-leukocyte aggregates, amplifying inflammation (Semple et al. 2011; Morrell et al. 2014). Thus, platelets have emerged as essential regulators of inflammation in various diseases (Semple et al. 2011).

Furthermore, thrombocytopenia may indicate inflammation as platelets are recruited to inflamed sites and adhere to white blood cells, thereby enhancing their activity and forming aggregates. Consequently, the circulating platelet count decreases (Seymour et al. 2016). Following recruitment from the bloodstream to sites of endothelial injury and inflammation, platelets were traditionally perceived as stationary cells. They adhere tightly and aggregate at the vessel wall, generating adhesive forces that activate fibrinogen. This activation subsequently triggers fibrin deposition and the formation of fibrin clots (Mackman et al. 2007). These consequences lead to thrombosis formation and interrupt blood flow which provides nutrition and oxygen to cells, ultimately culminating in cellular death (Chu 2005). Our findings demonstrated that pretreating rats with Clop before caused a marked reduction in fibrinogen and fibrin deposition compared to Genta-treated rats.

The present study also revealed the significance of apoptosis in Genta-induced renal injury. Genta administration led to a substantial increase in both caspase-3 and pro-apoptotic BAX expression, coupled with a notable decrease in anti-apoptotic Bcl-2 expression, suggesting activation of the apoptotic pathway in Genta-induced renal injury. Previous research has documented comparable results (Abouzed et al. 2021; Laorodphun et al. 2022; Nadeem et al. 2023). Conversely, Clop demonstrated renoprotective effects by attenuating the overexpression of caspase-3 and BAX while enhancing Bcl-2 expression. These findings are consistent with various investigative experiments highlighting the anti-apoptotic activity of Clop (Hu et al. 2011; Yip et al. 2012; Abouzed et al. 2021).

## Conclusion

The results of the investigation indicate that the activation of oxidative stress and apoptosis signaling pathways, as well as the process of coagulation system activation, are key contributors to the development of Genta-induced nephrotoxicity. Moreover, Clop exhibited robust nephron-protective effects against Genta-induced nephrotoxicity, attributed to its anticoagulant, antioxidant, anti-inflammatory, and anti-apoptotic properties.

## Data Availability

No datasets were generated or analysed during the current study.

## References

[CR1] Abdel-Bakky MS, Hammad MA, Walker LA, Ashfaq MK (2011) Tissue factor dependent liver injury causes release of retinoid receptors (RXR-α and RAR-α) as lipid droplets. Biochem Biophys Res Commun 410:146–151 21658367 10.1016/j.bbrc.2011.05.127

[CR2] Abdelkader RS-E, El-Beih NM, Zaahkouk SA, El-Hussieny EA (2022) Ameliorative effect of Eruca sativa seeds and its rutin on gentamicin-induced nephrotoxicity in male rats via targeting inflammatory status, oxidative stress and kidney injury molecule-1 (KIM-1)/cystatin c expression. Indones Biomed J 14:74–83

[CR3] Abdel-Raheem IT, Abdel-Ghany AA, Mohamed GA (2009) Protective effect of quercetin against gentamicin-induced nephrotoxicity in rats. Biol Pharm Bull 32:61–67 19122282 10.1248/bpb.32.61

[CR4] Abhirama BR, ShanmugaSundaram R, Raju A (2018) Amelioration of gentamicin-induced renal damage in rats by ethanol extract of the whole plant Biophytum sensitivum (linn.) DC. Int J Pharm Pharm Sci 10:130

[CR5] Abouzed TK, Sherif EAE, Barakat MES et al (2021) Assessment of gentamicin and cisplatin-induced kidney damage mediated via necrotic and apoptosis genes in albino rats. BMC Vet Res 17:1–9 34784920 10.1186/s12917-021-03023-4 PMC8594120

[CR6] Adikay S, Koganti B (2010) Effect of decoction of root bark of Berberis aristata against cisplatin ­ induced nephrotoxicity in rats. Int J Pharm Pharm Sci 2:51–56

[CR7] Ali BH, Al Za’abi M, Blunden G, Nemmar A (2011) Experimental gentamicin nephrotoxicity and agents that modify it: a mini-review of recent research. Basic Clin Pharmacol Toxicol 109:225–232 21599835 10.1111/j.1742-7843.2011.00728.x

[CR8] Al-Kuraishy HM, Al-Gareeb AI, Al-Nami MS (2020) Irbesartan attenuates gentamicin-induced nephrotoxicity in rats through modulation of oxidative stress and endogenous antioxidant capacity. Int J Prev Med 11:16 32175056 10.4103/ijpvm.IJPVM_567_18 PMC7050237

[CR9] Alsharidah M, Abdel-Moneim A-MH, Alsharidah AS et al (2021) Thymoquinone, but not metformin, protects against gentamicin-induced nephrotoxicity and renal dysfunction in rats. Appl Sci 11:3981

[CR10] Ansari MA, Raish M, Ahmad A et al (2017) Sinapic acid ameliorate cadmium-induced nephrotoxicity: in vivo possible involvement of oxidative stress, apoptosis, and inflammation via NF-κB downregulation. Environ Toxicol Pharmacol 51:100–107 28233699 10.1016/j.etap.2017.02.014

[CR11] Arulkumaran N, Turner CM, Sixma ML et al (2013) Purinergic signaling in inflammatory renal disease. Front Physiol 4:194 23908631 10.3389/fphys.2013.00194 PMC3725473

[CR12] Aurori M, Andrei S, Dreanca AI et al (2023) The nephroprotective effect of cornelian cherry (Cornus mas L.) and rowanberry (Sorbus aucuparia L.) in gentamicin-induced nephrotoxicity on Wistar rats with emphasis on the evaluation of novel renal biomarkers and the antioxidant capacity in correlation. Nutrients 15:4392 37892466 10.3390/nu15204392 PMC10609733

[CR13] Bai R, Fan J, Wang Y et al (2023) Protective effect of Cistanche deserticola on gentamicin-induced nephrotoxicity in rats. Chinese Herb Med 15:102–109 10.1016/j.chmed.2022.03.008 PMC9975639 36875447

[CR14] Balakumar P, Rohilla A, Thangathirupathi A (2010) Gentamicin-induced nephrotoxicity: do we have a promising therapeutic approach to blunt it? Pharmacol Res 62:179–186 20434560 10.1016/j.phrs.2010.04.004

[CR15] Basile DP, Anderson MD, Sutton TA (2012) Pathophysiology of acute kidney injury. Compr Physiol 2:1303 23798302 10.1002/cphy.c110041 PMC3919808

[CR16] Botros SR, Matouk AI, Anter A et al (2022) Protective effect of empagliflozin on gentamicin-induced acute renal injury via regulation of SIRT1/NF-κB signaling pathway. Environ Toxicol Pharmacol 94:103907. 10.1016/j.etap.2022.103907 35697188 10.1016/j.etap.2022.103907

[CR17] Burgucu HÇ, Olukman M, Coşkunsever D et al (2022) Palosuran in gentamicin-induced acute kidney injury in an experimental rat model. Turkish J Nephrol 31:127–133

[CR18] Campbell RE, Chen CH, Edelstein CL (2023) Overview of antibiotic-induced nephrotoxicity. Kidney Int Reports 8:2211–2225 10.1016/j.ekir.2023.08.031 PMC10658282 38025228

[CR19] Charles JP, Crouch SR (1977) Spectrophotometric and kinetics investigation of the Berthelot reaction for the determination of ammonia. Anal Chem 49:464–469. 10.1021/ac50011a034

[CR20] Chen J, Tang Y, Zhong Y et al (2022) P2Y12 inhibitor clopidogrel inhibits renal fibrosis by blocking macrophage-to-myofibroblast transition. Mol Ther 30:3017–3033 35791881 10.1016/j.ymthe.2022.06.019 PMC9481993

[CR21] Chu AJ (2005) Tissue factor mediates inflammation. Arch Biochem Biophys 440:123–132 16036212 10.1016/j.abb.2005.06.005

[CR22] Dacie JV, Lewis SM (2001) Practical haematology, 5 th. Churchill Livingstone, New York

[CR23] Elsisi AEE, Sokar SS, Shalaby MF, Abu-Risha SE-S (2021) Nephroprotective effects of febuxostat and/or mirtazapine against gentamicin-induced nephrotoxicity through modulation of ERK 1/2, NF-κB and MCP1. Expert Rev Clin Pharmacol 14:1039–1050 34030558 10.1080/17512433.2021.1933435

[CR24] Erçin U, Bilgihan A, Erkan AF, Yücel H (2019) New parameters of coronary artery diseases: oxidative stress markers. Tur Klin Biyokim Derg 17:48–55

[CR25] Esmon CT (2008) Reprint of crosstalk between inflammation and thrombosis. Maturitas 61:122–131 19437587 10.1016/j.maturitas.2008.11.008

[CR26] Evangelista V, Manarini S, Dell’Elba G et al (2005) Clopidogrel inhibits platelet-leukocyte adhesion and platelet dependent leukocyte activation. Thromb Haemost 94:568–577 16268474

[CR27] Ewees MGE-D, Abdel-Bakky MS, Bayoumi AMA et al (2021) Dabigatran mitigates cisplatin-mediated nephrotoxicity through down regulation of thrombin pathway Mohamed. J Adv Res 31:127–136. 10.1016/j.jare.2020.12.014 34194837 10.1016/j.jare.2020.12.014 PMC8240102

[CR28] Hadi NR, Mohammad BI, Ajeena IM, Sahib HH (2013) Antiatherosclerotic potential of clopidogrel: antioxidant and anti-inflammatory approaches. Biomed Res Int 2013:790263 24455725 10.1155/2013/790263 PMC3888675

[CR29] Hu H, Batteux F, Chéreau C et al (2011) Clopidogrel protects from cell apoptosis and oxidative damage in a mouse model of renal ischaemia–reperfusion injury. J Pathol 225:265–275 21630270 10.1002/path.2916

[CR30] Huang H, Jin WW, Huang M et al (2020) Gentamicin-induced acute kidney injury in an animal model involves programmed necrosis of the collecting duct. J Am Soc Nephrol JASN 31:2097 32641397 10.1681/ASN.2019020204 PMC7461673

[CR31] Ishimatsu T, Sasaki K, Kakuma T et al (2020) Serum interleukin-18 levels as a predictor for patients with genetic dysfunction of cytochrome P450 2C19 in dual antiplatelet therapy with clopidogrel. J Cardiol 76:479–486 32616329 10.1016/j.jjcc.2020.06.008

[CR32] Jia Z, Huang Y, Ji X et al (2019) Ticagrelor and clopidogrel suppress NF-κB signaling pathway to alleviate LPS-induced dysfunction in vein endothelial cells. BMC Cardiovasc Disord 19:1–7 31888640 10.1186/s12872-019-01287-1 PMC6936058

[CR33] Jiang X-L, Samant S, Lesko LJ, Schmidt S (2015) Clinical pharmacokinetics and pharmacodynamics of clopidogrel. Clin Pharmacokinet 54:147–166 25559342 10.1007/s40262-014-0230-6 PMC5677184

[CR34] Kanko M, Maral H, Akbas MH et al (2005) Protective effects of clopidogrel on oxidant damage in a rat model of acute ischemia. Tohoku J Exp Med 205:133–139 15673971 10.1620/tjem.205.133

[CR35] Kasap B, Türkmen M, Kiray M et al (2013) Effects of pentoxifylline on gentamicin-induced nephrotoxicity. Ren Fail 35:1376–1381. 10.3109/0886022X.2013.828359 23991939 10.3109/0886022X.2013.828359

[CR36] Khalaf N, Ashour R, Youssef M et al (2017) Potential neuroprotective effect of clopidogrel on aluminum chloride-induced Alzheimer disease in rats. Mansoura Med J 47:1–26

[CR37] Khalaf NEA, Ashour RH, Youssef MY et al (2018) Potential neuroprotective effect of clopidogrel on aluminum chloride-induced Alzheimer disease in rats. Mansoura Med J 47:1–8

[CR38] Kloss L, Dollt C, Schledzewski K et al (2019) ADP secreted by dying melanoma cells mediates chemotaxis and chemokine secretion of macrophages via the purinergic receptor P2Y12. Cell Death Dis 10:760 31591378 10.1038/s41419-019-2010-6 PMC6779894

[CR39] Kondera E, Bojarski B, Ługowska K et al (2020) Effects of oxytetracycline and gentamicin therapeutic doses on hematological, biochemical and hematopoietic parameters in Cyprinus carpio juveniles. Animals 10:2278 33287184 10.3390/ani10122278 PMC7761691

[CR40] Konosic S, Petricevic M, Ivancan V et al (2019) Intragastric application of aspirin, clopidogrel, cilostazol, and BPC 157 in rats: platelet aggregation and blood clot. Oxid Med Cell Longev 2019:9084643. 10.1155/2019/9084643 31976029 10.1155/2019/9084643 PMC6955135

[CR41] Kraus A, Grampp S, Goppelt-Struebe M et al (2016) P2Y2R is a direct target of HIF-1α and mediates secretion-dependent cyst growth of renal cyst-forming epithelial cells. Purinergic Signal 12:687–695 27565965 10.1007/s11302-016-9532-5 PMC5124009

[CR42] Krishnan S, Suarez-Martinez AD, Bagher P et al (2021) Microvascular dysfunction and kidney disease: challenges and opportunities? Microcirculation 28:e12661 33025626 10.1111/micc.12661 PMC9990864

[CR43] Kumar D, Singla SK, Puri V, Puri S (2015) The restrained expression of NF-kB in renal tissue ameliorates folic acid induced acute kidney injury in mice. PLoS One 10:e115947 25559736 10.1371/journal.pone.0115947 PMC4283964

[CR44] Laorodphun P, Cherngwelling R, Panya A, Arjinajarn P (2022) Curcumin protects rats against gentamicin-induced nephrotoxicity by amelioration of oxidative stress, endoplasmic reticulum stress and apoptosis. Pharm Biol 60:491–500 35188833 10.1080/13880209.2022.2037663 PMC8865128

[CR45] Lee D-H, Jacobs DR, Gross M et al (2003) γ-Glutamyltransferase is a predictor of incident diabetes and hypertension: the coronary artery risk development in young adults (CARDIA) study. Clin Chem 49:1358–1366. 10.1373/49.8.1358 12881453 10.1373/49.8.1358

[CR46] Lopez-Novoa JM, Quiros Y, Vicente L et al (2011) New insights into the mechanism of aminoglycoside nephrotoxicity: an integrative point of view. Kidney Int 79:33–45 20861826 10.1038/ki.2010.337

[CR47] Mackman N, Tilley RE, Key NS (2007) Role of the extrinsic pathway of blood coagulation in hemostasis and thrombosis. Arterioscler Thromb Vasc Biol 27:1687–1693 17556654 10.1161/ATVBAHA.107.141911

[CR48] Madhusudhan T, Kerlin BA, Isermann B (2016) The emerging role of coagulation proteases in kidney disease. Nat Rev Nephrol 12:94 26592189 10.1038/nrneph.2015.177 PMC4933505

[CR49] Matouk AI, Awad EM, Kamel AA et al (2023) Dihydromyricetin protects against gentamicin-induced nephrotoxicity via upregulation of renal SIRT3 and PAX2. Life Sci 336:122318 38035992 10.1016/j.lfs.2023.122318

[CR50] Mediero A, Wilder T, Reddy VSR et al (2016) Ticagrelor regulates osteoblast and osteoclast function and promotes bone formation in vivo via an adenosine-dependent mechanism. FASEB J 30:3887 27511945 10.1096/fj.201600616R PMC5067248

[CR51] Micklewright JJ, Layhadi JA, Fountain SJ (2018) P2Y12 receptor modulation of ADP-evoked intracellular Ca2+ signalling in THP-1 human monocytic cells. Br J Pharmacol 175:2483–2491 29574692 10.1111/bph.14218 PMC5980558

[CR52] Moore CS, Ase AR, Kinsara A et al (2015) P2Y12 expression and function in alternatively activated human microglia. Neuroimmunol Neuroinflamm 2:e80 10.1212/NXI.0000000000000080 PMC4370387 25821842

[CR53] Morrell CN, Aggrey AA, Chapman LM, Modjeski KL (2014) Emerging roles for platelets as immune and inflammatory cells. Blood J Am Soc Hematol 123:2759–2767 10.1182/blood-2013-11-462432 PMC4007605 24585776

[CR54] Nadeem RI, Aboutaleb AS, Younis NS, Ahmed HI (2023) Diosmin mitigates gentamicin-induced nephrotoxicity in rats: insights on miR-21 and -155 expression, Nrf2/HO-1 and p38-MAPK/NF-κ B Pathways. Toxics 11(1):48 10.3390/toxics11010048 PMC9865818 36668774

[CR55] Nassan MA, Soliman MM, Aldhahrani A et al (2021) Ameliorative impacts of Glycyrrhiza glabra root extract against nephrotoxicity induced by gentamicin in mice. Food Sci Nutr 9:3405–3413 34262702 10.1002/fsn3.2183 PMC8269671

[CR56] O’Connor S, Montalescot G, Collet J-P (2011) The P2Y 12 receptor as a target of antithrombotic drugs. Purinergic Signal 7:325–332 21710143 10.1007/s11302-011-9241-z PMC3166993

[CR57] Özcan O, Erdal H, Çakırca G, Yönden Z (2015) Oxidative stress and its impacts on intracellular lipids, proteins and DNA. J Clin Exp Invest 6:331–336

[CR58] Palta S, Saroa R, Palta A (2014) Overview of the coagulation system. Indian J Anaesth 58:515 25535411 10.4103/0019-5049.144643 PMC4260295

[CR59] Pavlović N, Kopsida M, Gerwins P, Heindryckx F (2020) Inhibiting P2Y12 in macrophages induces endoplasmic reticulum stress and promotes an anti-tumoral phenotype. Int J Mol Sci 21:8177 33142937 10.3390/ijms21218177 PMC7672568

[CR60] Perazella MA (2018) Pharmacology behind common drug nephrotoxicities. Clin J Am Soc Nephrol CJASN 13:1897 29622670 10.2215/CJN.00150118 PMC6302342

[CR61] Perazella MA, Rosner MH (2022) Drug-induced acute kidney injury. Clin J Am Soc Nephrol 17:1220–1233 35273009 10.2215/CJN.11290821 PMC9435983

[CR62] Pletz J, Enoch SJ, Jais DM et al (2018) A critical review of adverse effects to the kidney: mechanisms, data sources, and in silico tools to assist prediction. Expert Opin Drug Metab Toxicol 14:1225–1253 30345815 10.1080/17425255.2018.1539076

[CR63] Qu S, Dai C, Lang F et al (2019) Rutin attenuates vancomycin-induced nephrotoxicity by ameliorating oxidative stress, apoptosis, and inflammation in rats. Antimicrob Agents Chemother 63:10–1128 10.1128/AAC.01545-18 PMC6325182 30397060

[CR64] Randjelović P, Veljković S, Stojiljković N (2017) Gentamicin nephrotoxicity in animals: current knowledge and future perspectives. EXCLI J 16:388–399 28507482 10.17179/excli2017-165 PMC5427480

[CR65] Richard JH, Donald CC, James WW (1974) Clinical chemistry: principles and techniques, 2nd edn. Hagerstown, Md., Medical Dept., Harper & Row, New York

[CR66] Saeedavi M, Goudarzi M, Fatemi I et al (2023) Gentisic acid mitigates gentamicin-induced nephrotoxicity in rats. Tissue Cell 84:102191 37556917 10.1016/j.tice.2023.102191

[CR67] Samarghandian S, Azimi-Nezhad M, Mehrad-Majd H, Mirhafez SR (2015) Thymoquinone ameliorates acute renal failure in gentamicin-treated adult male rats. Pharmacology 96:112–117 26202209 10.1159/000436975

[CR68] Sanchez-Gonzalez PD, Lopez-Hernandez FJ, Perez-Barriocanal F et al (2011) Quercetin reduces cisplatin nephrotoxicity in rats without compromising its anti-tumour activity. Nephrol Dial Transplant 26:3484–3495 21602180 10.1093/ndt/gfr195

[CR69] Semple JW, Italiano JE, Freedman J (2011) Platelets and the immune continuum. Nat Rev Immunol 11:264–274 21436837 10.1038/nri2956

[CR70] Seymour CW, Liu VX, Iwashyna TJ et al (2016) Assessment of clinical criteria for sepsis: for the Third International Consensus Definitions for Sepsis and Septic Shock (Sepsis-3). Jama 315:762–774 26903335 10.1001/jama.2016.0288 PMC5433435

[CR71] Sharawy MH, Serrya MS (2020) Pirfenidone attenuates gentamicin-induced acute kidney injury by inhibiting inflammasome-dependent NLRP3 pathway in rats. Life Sci 260:118454 32950575 10.1016/j.lfs.2020.118454

[CR72] Sherif IO, Al-Mutabagani LA, Alnakhli AM et al (2015) Renoprotective effects of angiotensin receptor blocker and stem cells in acute kidney injury: involvement of inflammatory and apoptotic markers. Exp Biol Med 240:1572–1579 10.1177/1535370215577582 PMC4935333 25825359

[CR73] Simmons EM, Himmelfarb J, Sezer MT et al (2004) Plasma cytokine levels predict mortality in patients with acute renal failure. Kidney Int 65:1357–1365 15086475 10.1111/j.1523-1755.2004.00512.x

[CR74] Solini A, Usuelli V, Fiorina P (2015) The dark side of extracellular ATP in kidney diseases. J Am Soc Nephrol 26:1007–1016 25452669 10.1681/ASN.2014070721 PMC4413770

[CR75] Suárez-Álvarez B, Liapis H, Anders H-J (2016) Links between coagulation, inflammation, regeneration, and fibrosis in kidney pathology. Lab Investig 96:378–390 26752746 10.1038/labinvest.2015.164

[CR76] Sutton TA, Fisher CJ, Molitoris BA (2002) Microvascular endothelial injury and dysfunction during ischemic acute renal failure. Kidney Int 62:1539–1549 12371954 10.1046/j.1523-1755.2002.00631.x

[CR77] Tu X, Chen X, Xie Y et al (2008) Anti-inflammatory renoprotective effect of clopidogrel and irbesartan in chronic renal injury. J Am Soc Nephrol JASN 19:77 18045851 10.1681/ASN.2007020160 PMC2391039

[CR78] Tvedten H, Raskin RE (2012) Leukocyte disorders. Small Anim Clin Diagnosis by Lab Methods 63:91

[CR79] Verma SK, Molitoris BA (2015) Renal endothelial injury and microvascular dysfunction in acute kidney injury. In: Seminars in nephrology. Elsevier, pp 96–107 10.1016/j.semnephrol.2015.01.010 PMC4476528 25795503

[CR80] Wu B, Yu J, Luo Y et al (2022) An albumin-enriched nanocomplex achieves systemic delivery of clopidogrel bisulfate to ameliorate renal ischemia reperfusion injury in rats. Mol Pharm 19:3934–3947 36067352 10.1021/acs.molpharmaceut.2c00401

[CR81] Yahyazadeh R, Baradaran Rahimi V, Yahyazadeh A et al (2021) Promising effects of gingerol against toxins: a review article. Biofactors 47:885–913 34418196 10.1002/biof.1779

[CR82] Yeung J, Li W, Holinstat M (2018) Platelet signaling and disease: targeted therapy for thrombosis and other related diseases. Pharmacol Rev 70:526–548 29925522 10.1124/pr.117.014530 PMC6013590

[CR83] Yip H-K, Yang J-L, Chua S et al (2012) Combination of cilostazol and clopidogrel attenuates Rat critical limb ischemia. J Trans Med 10:164 10.1186/1479-5876-10-164 PMC3479044 22897925

[CR84] Zarei B, Elyasi S (2022) Saffron nephroprotective effects against medications and toxins: a review of preclinical data. Iran J Basic Med Sci 25:419 35656071 10.22038/IJBMS.2022.61344.13570 PMC9150802

[CR85] Zhu T, Xu Y, Dong B et al (2011) β-elemene inhibits proliferation of human glioblastoma cells through the activation of glia maturation factor β and induces sensitization to cisplatin. Oncol Rep 26:405–413 21519795 10.3892/or.2011.1276

